# Evolutionary adaptations of TRPA1 thermosensitivity and skin thermoregulation in vertebrates

**DOI:** 10.1016/j.isci.2025.113369

**Published:** 2025-08-14

**Authors:** Gabriel E. Bertolesi, Neda Heshami, Sarah McFarlane

**Affiliations:** 1 Hotchkiss Brain Institute and Alberta Children’s Hospital Research Institute, Department of Cell Biology and Anatomy, University of Calgary, Calgary, AB, Canada

**Keywords:** Skin temperature, Evolutionary ecology, Evolutionary developmental biology

## Abstract

Skin pigmentation contributes to thermoregulation in ectothermic vertebrates, while homeotherms rely on insulation such as feathers, fur, or blubber. Heat-sensing in vertebrates largely depends on transient receptor potential (TRP) channels, with TRPA1 showing evolutionary shifts in sensitivity. The exploration of a role for TRP channels in skin physiology has largely focused on human pigmentation and overlooked the evolution of different thermoregulatory structures in the integument of distinct vertebrates. We investigated heat-induced skin darkening in the ectotherm *Xenopus laevis* and found TRPA1 mediates melanosome dispersion. Conversely, TRPA1 mediates cold sensation in rodents and UV-induced tanning in humans. In Euarchontoglires, a switch to TRPA1 cold thermal sensitivity correlates with a change in an essential amino acid (V→G878). Aquatic mammals (manatees, whales) that rely on blubber for insulation show reduced TRPA1 selection pressure as compared to their terrestrial relatives. Our findings highlight TRPA1’s adaptive evolution across vertebrates, linking thermal sensing to integumentary specialization.

## Highlights

• In *Xenopus laevis,* TRPA1 heat thermal sensitivity mediates melanosome dispersion
• TRPA1 heat sensitivity in ectotherms changed when homeotherms evolved
• A V→G878 shift in Glires links cold sensitivity with phylogenetic divergence
• Higher TRPA1 mutation rates in aquatic mammals align with blubber-based adaptation

## Graphical abstract

**Figure:**
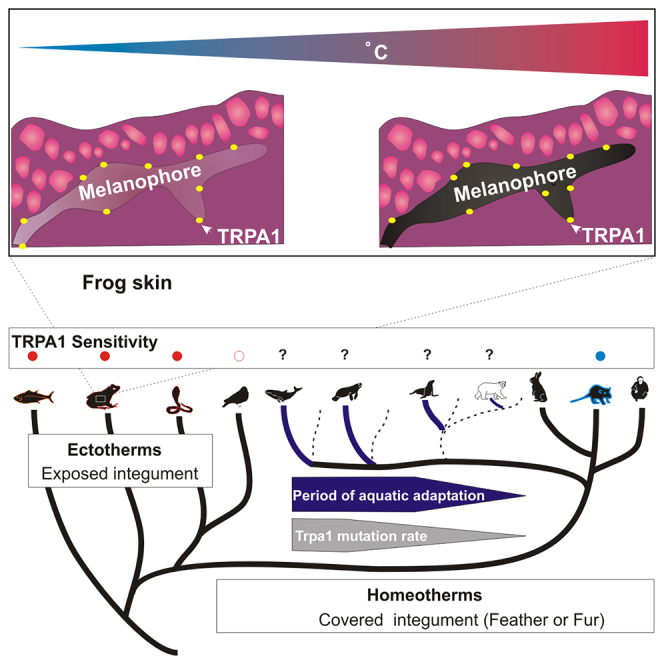

## Introduction

Skin color and/or reflectance alterations play a vital role in regulating body temperature to sustain life.^1^^,^^2^ These mechanisms are crucial for survival of poikilothermic vertebrates, but are less relevant for birds and mammals, where feathers and fur in their integument emerged as integral traits for insulation during the evolution of endothermy.^3^ Members of the transient receptor potential (TRP) non-selective cation channel family are considered the classical thermosensors of vertebrates, although their range of temperature sensitivity differs drastically between lineages.^4^^,^^5^ This is particularly true of TRPA1 that is a heat sensor in poikilotherm vertebrates [ea. amphibians and reptiles^6^^,^^7^^,^^8^, a cold sensor in rodents, and temperature insensitive in primates.^9^^,^^10^^,^^11^ Unknown is the role of TRPA1 in regulating skin color and/or reflectance alterations, and the extent to which the physiology of skin pigmentation and the evolutionary divergence of TRPA1 temperature sensitivity are linked.

Variations in color change and reflectance are generated by the movement of pigmented or reflective organelles located in dermal chromatophores. Eight types of chromatophores containing absorptive or reflective pigments are present in vertebrates.^12^ *Xenopus laevis* tadpoles offer an advantageous model for studying skin color change, as the melanin pigment containing melanophores are the main mediators of color change through organelle dispersion to cause skin darkening and aggregation to induce skin lightening.^13^^,^^14^ In this species, Trpm8 in melanophores senses cold to induce pigment aggregation and skin lightening.^15^ TRPM8, as a cold sensor, is relatively conserved in vertebrates, though it underwent significant evolutionary change in thermal activity during the transition of organisms from water to land.^16^ Unknown is whether melanophores express a heat sensor to induce melanosome dispersion and skin darkening under warmer conditions to counteract the skin lightening induced by cold through Trpm8.

Trpv1 and Trpa1 are both candidates for the heat sensor to regulate skin color in poikilothermic vertebrates. These channels are often co-expressed, as per in dorsal root ganglion neurons.^17^ Depending on the species, however, the temperature sensitivity of TRPA1 differs. For instance, TRPA1 is a cold sensor in mouse, rat and other mammals,^5^^,^^10^^,^^18^ and a heat sensor in amphibians and reptiles.^7^^,^^8^ Human TRPA1 thermal sensitivity is controversial (reviewed in^5^^,^^19^^,^^20^). However, when identical experimental conditions are used to determine sensitivity, TRPA1 functions as a cold sensor in rodents, as a heat sensor in reptiles, and is insensitive in humans.^6^^,^^9^ Trpa1 actually forms a dimer with Trpv1 to mediate thermal sensitivity in a group of embryonic turtle terminal sensory neurons^21^ to control heat-induced thermotaxic behavior.^22^ These two channels are co-expressed in melanocytes of human skin,^23^ the mammalian equivalent of poikilothermic melanophores. Data from humans suggests that ultraviolet (UV) light, rather than the temperature activates TRPA1 to darken the skin through melanin synthesis,^24^^,^^25^ although the mechanisms involved are unknown. These findings suggest that evolutionary changes in TRPA1 altered its physiological role in skin pigmentation. The conservation of TRPV1 as a heat sensor throughout vertebrate evolution^4^ may also have influenced the function of TRPA1 as a thermosensor in pigmentary physiology. Here, we find that warm temperatures induce skin darkening in *Xenopus laevis* tadpoles and examine the expression of Trpv family members and Trpa1 in the skin and melanophores to identify the channel responsible for heat sensing.

While thermoregulation is a critical physiological function shared between melanin-mediated coloration and TRPA1, significant evolutionary events likely altered their respective roles in maintaining thermal homeostasis. First, by conquering the land, terrestrial ectothermic reptiles gained the ability to adjust their skin pigmentation to regulate body temperature through the reflection or absorption of irradiated energy.^2^^,^^26^ Second, in extinct marine reptiles, the melanization data from fossilized skin suggest a contribution of pigmentation to the adaptation to cold-water habitats.^27^^,^^28^^,^^29^ Pigmentation mechanisms became less crucial in homeotherms with feathers or fur, as the advent of endothermy sculpted their skin physiology. Hominids, however, lost body hair and gained sweat glands during the evolution of bipedalism as a more efficient system to dissipate heat.^30^^,^^31^ Interestingly, in humans, melanin synthesis in skin melanocytes is strongly induced by UV light rather than temperature, a pigmentation physiological response that requires TRPA1 activation.^25^^,^^32^^,^^33^ Whether changes in the thermal activation of TRPA1 in vertebrate melanophores/melanocytes are linked to evolutionary adaptations in thermoregulation or skin physiology remains unknown.

To explore a possible link, we first analyzed the thermosensor role for Trpa1 in the skin pigmentation of ectotherms, turning to *Xenopus* laevis as we and others have shown it is an excellent model to identify the molecular mechanisms that underlie the regulation of skin pigmentation.^14^^,^^34^ Second, we conducted an evolutionary study, comparing the thermal sensitivity of TRPA1 in related extant species of chordates based on electrophysiological data from the literature. Our analysis focused on the role of the integument for thermoregulation. For instance, while altering skin color mediates thermoregulation in ectotherm vertebrates,^1^^,^^2^ fur and feathers were positively selected for thermal insulation during the evolution of thermoregulation in endotherms.^3^ Marine mammals possess thick layers of blubber, and their thermoregulation primarily depends on cardiovascular adjustments rather than skin pigmentation.^35^ Blubber acts as both a thermal barrier and being highly vascularized allows the adjustment of blood flow to the skin and extremities to conserve or dissipate heat as needed.^35^^,^^36^ Mammals re-entered the marine environment at least seven times throughout evolution, with extant species originating from five of these events.^37^ Notably, the anatomical and physiological changes in the integument differ for each instance of re-entry, reflecting evolutionary adaptations. In general, early adapted species [e.g. Sirenia (manatees and dugongs) and Cetaceans (whales and dolphins)] have hairless and thick skin with a blubber layer. A more recently adapted lineage, the pinnipeds (seals and sea lions), relies on both blubber and fur and behavior for thermoregulation, while polar bears possess a dense hair-covered fur for thermoregulation.^38^^,^^39^^,^^40^^,^^41^^,^^42^ Thus, these marine mammals provide an excellent model to examine whether evolutionary changes in thermoregulatory mechanisms between them and their terrestrial relatives are reflected in the mutation rate of TRPA1.

We show that heat disperses melanosomes, darkening the skin of *Xenopus laevis* and melanophores *in vitro,* in a manner that we find through pharmacology and siRNA knockdown depends on Trpa1 as the heat sensor. Our investigation of the evolution of TRPA1 thermosensation in chordates shows interesting correlations regarding changes in TRPA1 thermal sensitivity. First, we find that TRPA1 acts as a heat sensor in organisms with uncovered integument (fish, amphibians, and reptiles). Second, in Euarchontoglires, differences in cold thermal sensitivity between certain rodent lineages [Myomorphs (Mice and rats)] with respect to Sciuromorphs (squirrels), Lagomorphs (rabbits, hares, and pikas) and Primates^43^ correlates with the presence of an essential amino acid (G > V878), and it is explained by the evolutionary divergence between these lineages. Finally, our comparative analysis of TRPA1 in mammals that returned to a marine habitat during evolution shows that the Sirenians and Cetaceans, which adapted by losing hair and developing thick blubber, exhibit a higher mutational rate in the protein region responsible for thermoregulation as compared to their terrestrial relatives, suggestive of less thermal positive selection pressure. Collectively, our data reveal a functional role for TRPA1 both in skin pigmentation and more broadly in the various thermoregulatory adaptations that appeared throughout chordate evolution, from the development of fur and feathers to adaptations for marine environments.

## Results

### Determination of an ecologically relevant heat condition for *Xenopus laevis* tadpoles

To determine an ecologically relevant heating paradigm for *Xenopus laevis*, we analyzed the average temperatures recorded over the last 30 years at three national parks in southern Africa (Etosha in Namibia, Kruger in South Africa, and Hwange in Zimbabwe). These national parks are located in regions where *Xenopus laevis* is native.^44^ We examined the temperatures during November and December, which exhibit a large temperature fluctuation between cold (mean daily minimum and cold nights) and hot (mean daily maximum and hot days) conditions (Table 1). The daily temperature fluctuations during these two months oscillate between 37°C (hot) and 16°C (cold) (Table 1). However, since *Xenopus* is an aquatic species, the recorded heat temperature variations are likely overestimated, as water acts as a thermal buffer. Indeed, stage 43/44 tadpoles did not survive at 37°C but remained viable and active at 32°C (Table 1). Therefore, we analyzed the skin pigmentation response to temperature fluctuations between 16°C and 32°C.

**Table 1:** Temperature selection (Degrees Celsius) based on the November and December temperatures registered for three national parks located in southern Africa

		Cold		Hot	
		Mean daily min.	Cold nights	Mean daily max.	Hot days
Etosha (Namibia)	Nov.	18	12	36	40
	Dec.	20	14	35	39
Kruger (South Africa)	Nov.	19	14	30	38
	Dec.	20	15	30	37
Hwange (Zimbawe)	Nov.	23	18	35	39
	Dec	22	18	33	38
Average Temperature	–	20.3	15.2	33.2	38.5
	Tested	16	–	37	a
Temperature	Selected/used	16	–	32	–

a Indicates embryos do not survive at this temperature.

### Heat induces a fast, reversible, and systemic skin darkening

**Figure 1:**
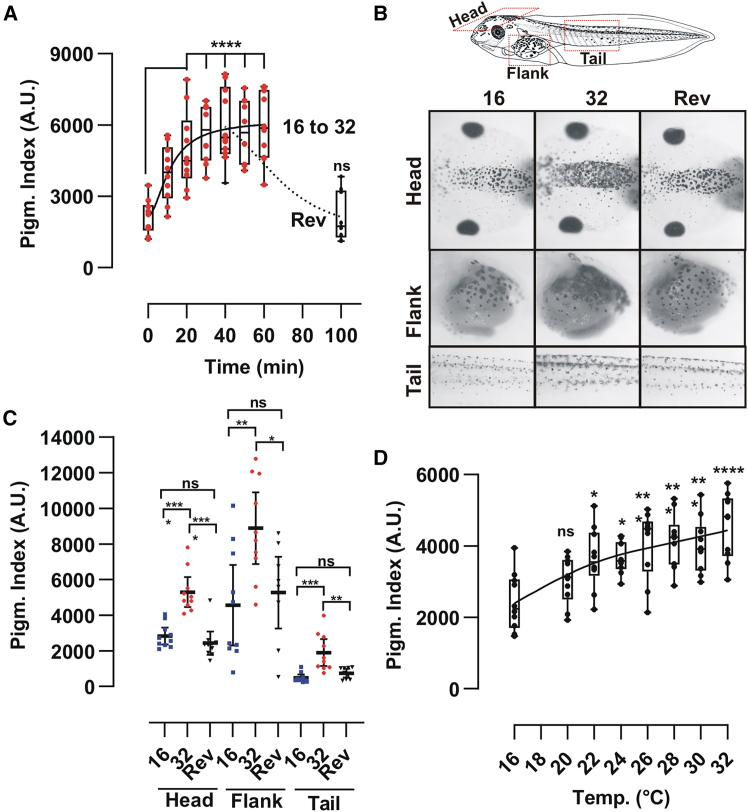
Darkening of skin color triggered by warm temperatures is temperature dependent, fast, reversible, and a systemic response of skin melanophores (A) Time dependent change in pigmentation index quantified from digital images of the dorsal head of *Xenopus* stage 43/44 tadpoles moved from 16°C to 32°C degrees; [individual data points (*n* = 10 embryos) and a boxplot (25^th^ to 75^th^ percentile) are represented; A representative study from three (*N* = 3) independent experiments is shown]. (B and C) Schematic of stage 42/43 tadpole and representative pictures (B) and pigmentation index (C) from the dorsal head, lateral belly (flank) and the tail of tadpoles at 16°C (16; blue dots), moved to 32°C for 45 min (32; red dots), or switched back to 16°C after being for 45 min at 32°C (Reversibility; Rev; black dots); [Horizontal bar represents the mean ±95% Confidence Interval; *n* = 9 embryos; A representative of three (*N* = 3) independent experiments is shown)]. (D) Temperature-dependent increase in the head pigmentation index of tadpoles moved from 16°C to the indicated temperatures. [individual data points (*n* = 8/9 embryos) and a boxplot (25^th^ to 75^th^ percentile) are represented; A representative study of three (*N* = 3) independent experiments is shown].

We asked if *Xenopus* tadpoles darken their skin in response to warmer temperatures (16°C–32°C). This paradigm is opposite to that used previously in our laboratory, where cooling conditions (24°C–6°C) induced skin lightening mediated by Trpm8.^15^ Importantly, we took two approaches to ensure temperature was the only variable that changed in our experiments. First, the analysis of pigmentation variation by temperature was always performed at the approximate middle of the light phase (Zeitgeber +5 to +7) since skin color changes in a daily circadian manner.^45^ Second, the color of the surface (white) and the overhead illumination (approximately 1000 lux) were always the same to avoid pigmentation changes induced by background adaptation.^14^^,^^46^ We observed that switching tadpoles from 16°C to 32°C induced rapid skin darkening that reached a maximum response after 40–45 min and resulted in an increased pigmentation index (Figure 1A). This response was reversible, with lightening occurring when tadpoles were moved back to 16°C (Figure 1A; Rev). The darkening in warm conditions and the reversibility were true for melanophores located in different body regions, including the head, belly, and tail (Figures 1B and 1C), suggesting a systemic response. Finally, we observed that the pigmentation index adjusted to the environmental temperature. When we moved tadpoles gradually from 16°C to 32°C over a 1-h period and measured the head pigmentation index, gradual skin darkening was detected in a temperature-dependent manner (Figure 1D). Of note, the smallest temperature change that was needed to significantly impact skin color varied between hatches; We detected a significant response as low as 22°C (in 1 out of 3 experiments), with the response continuing to progress until the maximum tested temperature (32°C) (Figure 1D).

Together, these results demonstrate that warm conditions induce a rapid, temperature-dependent, and systemic darkening of the skin, which is reversible when the temperature is reduced.

### Cultured melanophores disperse melanosomes when the temperature rises

**Figure 2:**
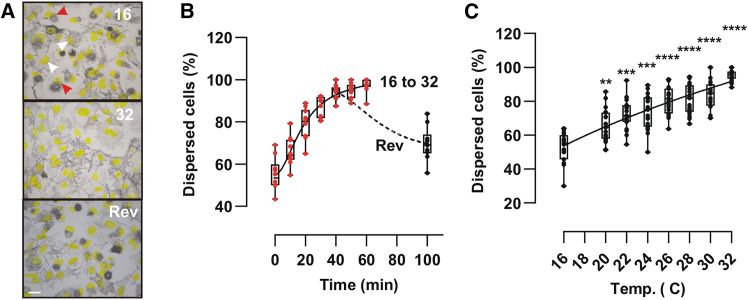
Warm temperature induces melanosome dispersion in melanophores *in vitro* (A) Representative brightfield images of MEX cell melanophores at 16°C with approximately 50% of the cells showing melanosome dispersion (white arrows) or aggregation (red arrows) at 16°C or after 1 h at 32°C. Cells returned to 16°C (reversibility; Rev) aggregate melanosomes. DAPI stained nuclei are yellow. Scale bar = 10 μm. (B and C) Time (B) and temperature dependent (C) increase of the melanosome dispersion of MEX cells moved from 16°C to 32°C degrees [individual data points (*n* = 6 pictures) and a boxplot (25^th^ to 75^th^ percentile) are represented; A representative study from three (*N* = 3) independent experiments is shown].

The systemic response observed *in vivo* led us to hypothesize a cell-autonomous response where the melanophores themselves were the sensors of high temperatures. To test this hypothesis, we switched to a melanophore cell line obtained from *X. laevis* embryos^47^ and analyzed their melanosome aggregation/dispersion in response to heat. To mimic the *in vivo* condition, the *in vitro* system was set up in 35 mm dishes with growth medium without phenol red [mimicking the colorless Modified Marc’s Ringer (MMR) solution used *in vivo*], with identical light conditions, since the aggregation and dispersion of melanophores *in vitro* are affected by light.^45^^,^^48^ As we showed previously, approximately half the population of MEX cells grown with 5% fetal calf serum-supplemented media exhibited melanosome dispersion (Figure 2A, 16°C; white arrows), with the remaining cells showing aggregated melanosomes (Figure 2A, 16°C; red arrows). Exposing the cells to a higher temperature of 32°C induced a time- and temperature-dependent dispersion of melanosomes that reached a maximum after approximately 45 min (Figures 2A–2C), and which was reversible (Figure 2B). These results show that the melanosome dispersion induced by heat is mediated via a cell-autonomous mechanism.

### Identification and expression of *trpv* and *trpa* family members in *Xenopus laevis* melanophores

To test the idea that the rapid change in skin pigmentation induced by heat in amphibians is mediated by a Trp channel(s), we first identified all *trpv* and *trpa* family members in the *Xenopus laevis* genome and analyzed their expression in melanophores. *X. laevis* is an allotetraploid species that arose via the interspecific hybridization of diploid progenitors around 18 million years ago.^49^ Consequently, its duplicated genome contains 36 chromosomes, consisting of 18 long (L) and 18 short (S) homologous chromosomes.^49^ While several of the duplicated genes have remained distinct, others were reduced to a single copy due to pseudogenization.

**Figure 3:**
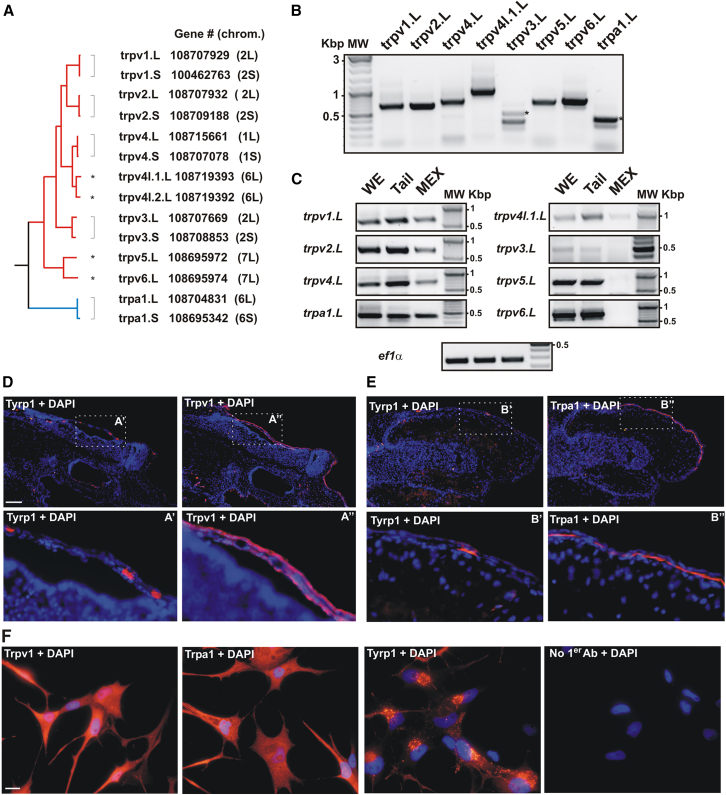
Identification and expression of *trpv* and *trpa* family members in *Xenopus laevis* melanophores (A) Molecular phylogenetic analysis by the maximum likelihood method of Trpv and Trpa family members (not to scale) of the predicted proteins from the genes found in the *X. laevis* genome. The name, gene number and chromosome localization are indicated. Brackets denote genes duplicated and maintained on the long (L) and short (S) chromosomes, with single copy genes indicated with an asterisk. (B and C) Representative RT-PCR analysis of mRNA expression in stage 43/44 whole embryos (B), and for isolated tails and MEX cells (C). The expressed mRNAs (L or S) identified after sequencing are indicated. Representative example of 3 independent samples; *N* = 3. (D–F) Immunohistochemistry for Trpv1 (D, F) or Trpa1 (E, F) and the Tyrosinase related protein 1 (Tyrp1) in consecutive sections (D, E), and in MEX cells (F). DAPI staining (blue) was used to visualize cell nuclei and facilitate the overlapping of adjacent sections in D and E. Boxed areas are shown enlarged (D′ or E′ and D″ or E″). Scale bar in D = 100 μm. Immunolabel indicates co-localization in melanophores (M) of the skin (S) of Tyrp1 (arrows) with the Trp channels. Note, adjacent sections were used as all antibodies were generated in rabbits. Note that Tyrp1 is localized to melanosomes while the Trp channels are in the plasma membrane. Scale bar in F = 10 μm.

We found that most of the genes from the *trpv* family remained duplicated, except for *trpv5* and *trpv6*, each with only one copy located on the same chromosome (7L) (Figure 3A). Interestingly, two additional genes with unknown functions are found in the *X. laevis* genome, named *trpv4-like 1* and *trpv4-like 2*, which show high homology at the amino acid level to the predicted Trpv4 proteins (Figure 3A). Previous structural and molecular analysis of the Trpv4 amino acid sequence from the western clawed frog suggests that Trpv4 retained its original function, while the Trpv4-like versions diversified, with slightly different properties.^50^ Nevertheless, these *trpv4-like* genes likely originated from intrachromosomal duplication before the generation of the *X. laevis* species, as both are present in the *X. tropicalis* genome and located on the same chromosome (6L). Finally, the *trpa1* genes were also located on chromosome 6 and remained duplicated in the *X. laevis* genome (Figure 3A).

Next, we determined which *trpa1* and *trpv* family members were expressed in whole stage 43/44 tadpoles by RT-PCR. While primers were designed to amplify both chromosomal variants (L and S) (Table S1), cloning and sequencing of the corresponding amplicons showed the exclusive expression of genes located on the L chromosomes (Figure 3B). To assess if melanophores express *trpa1* and *trpv* mRNAs, we also analyzed expression in isolated tails, as an *in vivo* proxy for skin, and in MEX cells. All the mRNAs were expressed in the tails, while MEX cells expressed *trpv1*, *trpv2*, *trpv4*, and *trpa1*, but little or no *trpv4-like 1*, *trpv3*, *trpv5*, and *trpv6* mRNA (Figure 3C). Finally, we determined for Trpv1 and Trpa1, where antibodies were available, whether the proteins were expressed by skin melanophores. We performed on tadpole frozen sections immunohistochemistry for either Trpv1 or Trpa1, with adjacent sections immunostained for Tyrosinase-related protein 1 (Tyrp1) to identify melanophores. Adjacent sections were analyzed since all antibodies were produced in rabbits. Both Trpv1 and Trpa1 were detected throughout the epidermis, including in Tyrp1-positive melanophores (Figures 3D and 3E). In support, Trpv1 and Trpa1 were also expressed by MEX cells (Figure 3F).

### Trpa1 is the heat sensor for melanophore dispersion with warm temperatures

To identify the TRP channel responsible for pigment dispersion by heat, we focused on the Trp channels expressed in both the tails and MEX cells: *trpv1*, *trpv2*, *trpv4*, and *trpa1*. Each Trp channel has a distinct range of thermal sensitivity and activation by chemicals,^4^^,^^5^^,^^51^ therefore we initially performed a pharmacological study. We excluded Trpv2 from our analysis because the literature argues against this channel playing a role in thermosensation at environmentally relevant temperatures; In mammals, TRPV2 is only activated by extreme heat (52°C) and *Trpv2* deficient mice display normal thermosensation.^52^ Further, Trpv2 lacks specific agonists, with the most commonly used TRPV2 agonist, 2-Aminoethoxydiphenyl borate (2-APB), also modulating other TRP channels.^53^ Thus, we analyzed the effect of specific agonists and antagonists of Trpv1, Trpv4 and Trpa1 on the melanosome dispersion of tadpoles *in vivo* and MEX cells *in vitro*.

**Figure 4:**
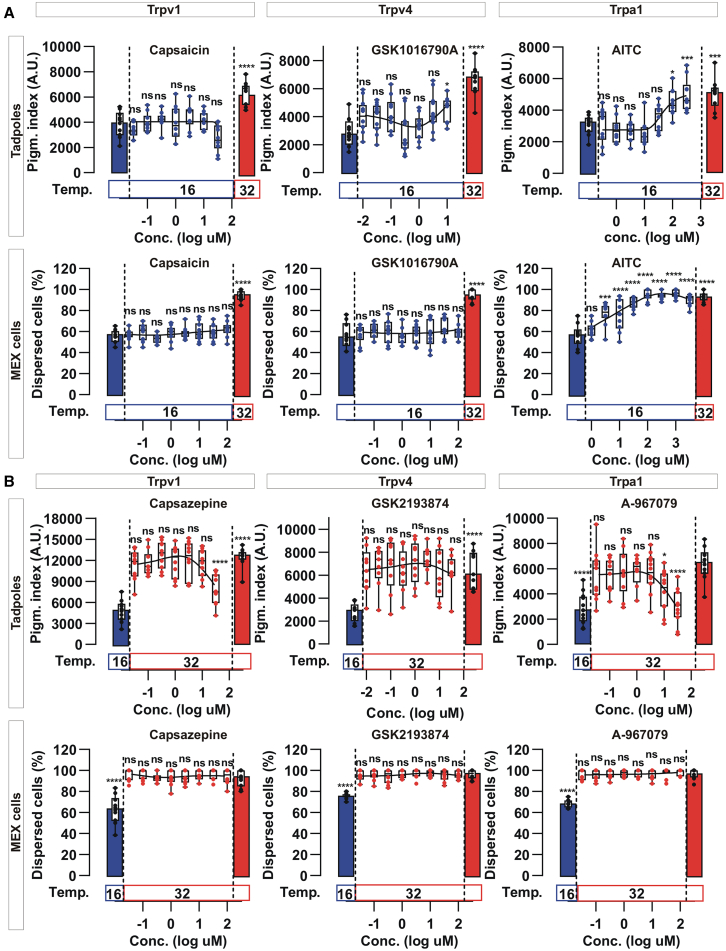
Melanosome dispersion is affected by a Trpa1 agonist and antagonist (A and B) Tadpoles (Stage 43/44) or MEX cells were treated with agonists (A) or antagonists (B) against the indicated Trp channels. The agonists were added at 16°C and maintained at that temperature, while antagonists were added at 16°C, and the embryos were then switched to 32°C for 45 min. Data points (*n* = 9 embryos or *n* = 6 MEX cells pictures) and a boxplot (25^th^ to 75^th^ percentile) are represented; A representative study from three (*N* = 3) independent experiments is shown, with Capsaicin (*N* = 2). Statistical significances for the different compounds are indicated against the control without the drug at the same temperature. ns; non significant. ∗; *p* < 0.05. ∗∗; *p* < 0.01.∗∗∗; *p* < 0.001. ∗∗∗∗; *p* < 0.0001.

Capsaicin (Figure 4A) and Piperine (Figure S1), the Trpv1 active compounds found in chili pepper and black pepper, respectively, did not darken the skin of tadpoles when added at 16°C. They also did not change melanosome distribution in MEX cells (Figures 4A and S1), suggesting Trpv1 is not involved in skin darkening. TRPV4 is expressed in mammalian melanocytes,^54^ yet neither the pigmentation index *in vivo* nor the dispersion of melanosomes *in vitro* was significantly affected by GSK1016790A, a potent and selective TRPV4 agonist (Figure 4A). Additionally, Trpv1 (capsazepine) and Trpv4 (GSK2193874) antagonists failed to block the melanosome dispersion induced by switching tadpoles and MEX cells from 16°C to 32°C, at doses that were not toxic to the embryo (Figure 4B). These results suggest that Trpv1 and Trpv4 are not the thermosensors involved in melanosome dispersion.

Although the thermal sensitivity of TRPA1 differs among distantly related animal species, chemical sensitivity to electrophilic compounds, particularly the Trpa1 agonist, Allyl isothiocyanate (AITC; oil of mustard), is conserved; demonstrated through electrophysiological studies in zebrafish,^55^ frogs,^7^ reptiles,^7^^,^^56^ birds,^57^ rodents, and humans.^9^ Interestingly, AITC induced a dose-dependent dispersion of melanosomes *in vivo* and *in vitro* (Figure 4A), suggesting Trpa1 activation induces a similar dispersion response to that seen with heat. To confirm a role for Trpa1, we next asked if a Trpa1 antagonist blocked the melanosome dispersion induced by switching tadpoles and MEX cells from 16°C to 32°C. Identifying a useful TRPA1 antagonist is difficult, in that the known antagonists show striking species-specific differences. For example, A-967079, a potent mammalian antagonist,^58^ acts as an agonist in chicken and has no effect in *X*. *tropicalis* heterologous overexpression systems.^59^^,^^60^^,^^61^ Interestingly, we observed a partial antagonist response in *X. laevis* tadpoles (Figure 4B), but were uncertain if this did not reflect toxicity (Figure 4B). *In vitro*, however, A-967079 showed no effect on the melanosome dispersion of MEX cells (Figure 4B).

**Figure 5:**
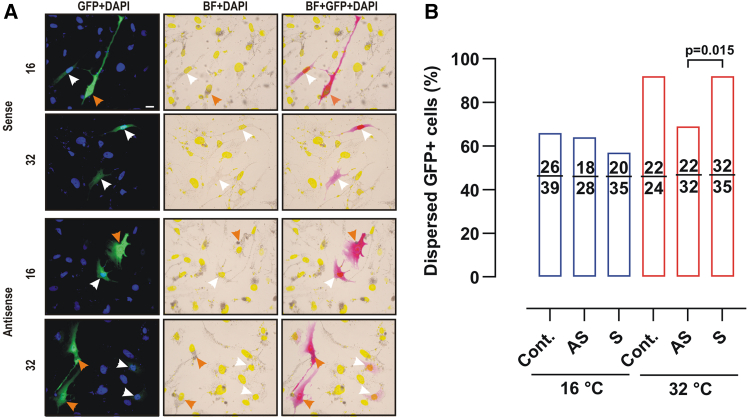
Melanosome dispersion induced by temperature is mediated by Trpa1 (A) MEX cells transfected with a GFP-expression vector (control) or siRNA oligonucleotides, sense (S) or antisense (AS) against Trpa1, together with GFP in a 5:1 ratio. GFP-expressing cells had aggregated (orange arrow) or dispersed (white arrow) pigment. Representative images show the merge between GFP (pink), DAPI (yellow), and brightfield (BF). Scale bar = 10 μm. (B) Quantification of melanosome dispersion in GFP-expressing cells observed by shifting cells from 16°C to 32°C for 1 h after 48 h of transfection. Data are expressed as the percentage of GFP positive cells with dispersed melanosomes relative to the total number of GFP positive MEX cells (*N* = 3; *n* = 20 pictures). Numbers in the bars indicate the counted cells. *p* value indicates a Fisher’s exact test comparing S and AS at 32°C.

Thus, to confirm that Trpa1 activation causes melanosome dispersion, we turned instead to siRNA-mediated knockdown of Trpa1 in MEX cells. The expectation would be that *trpa1* siRNA should block the dispersion response to heat. Preliminary assays on MEX cells showed a transfection efficiency of less than 20% when using GFP expression vectors, making it difficult to determine the siRNA efficacy on the entire MEX cell population in culture. Therefore, we co-transfected siRNA with GFP at a 5:1 ratio using micelles of lipofectamine 2000 and analyzed only the heat response of GFP-expressing cells. GFP+ melanophores expressing sense-siRNA (S) and cells transfected only with a GFP control plasmid showed normal melanosome dispersion in response to heat (Figures 5A and 5B). In contrast, GFP+ melanophores failed to disperse their melanophores when switched from 16°C to 32°C when Trpa1 was silenced with antisense-siRNA (AS). These results argue strongly that Trpa1 activation in *Xenopus* ectotherms is triggered by temperature and induces melanophore darkening.

### Melanogenesis and Trpa1 thermal sensitivity are linked to the evolution of thermoregulation

These data indicate that Trpa1 functions in skin pigmentation responses to heat in an ectotherm. These results differ from findings in hairy rodents where the channel is particularly sensitive to mechanical and noxious chemical cutaneous stimuli rather than temperature.^11^ Moreover, TRPA1 thermosensitivity to cold rather than heat was demonstrated by using TRPA1 deficient mice and rats.^10^^,^^62^ Finally, while TRPA1 does promote skin pigmentation in humans, it is UV light rather than temperature that induces tanning through melanin synthesis.^25^^,^^32^ Taken together these data suggest an evolutionary shift in the role of TRPA1 in skin physiology. Melanin and Trpa1 likely share an important thermoregulatory role in ectotherms^2^^,^^26^ and extinct aquatic tetrapods that used skin pigmentation to manage their physiology for survival in cold environments.^27^^,^^28^^,^^29^ In contrast, the function of TRPA1 as a thermosensor may have co-evolved with the advent of integumentary coverings such as hair and feathers for thermoregulation. If true, changes in organismal thermoregulation/thermal-sensitivity and skin physiology should be reflected in the TRPA1 molecular structure.

**Figure 6:**
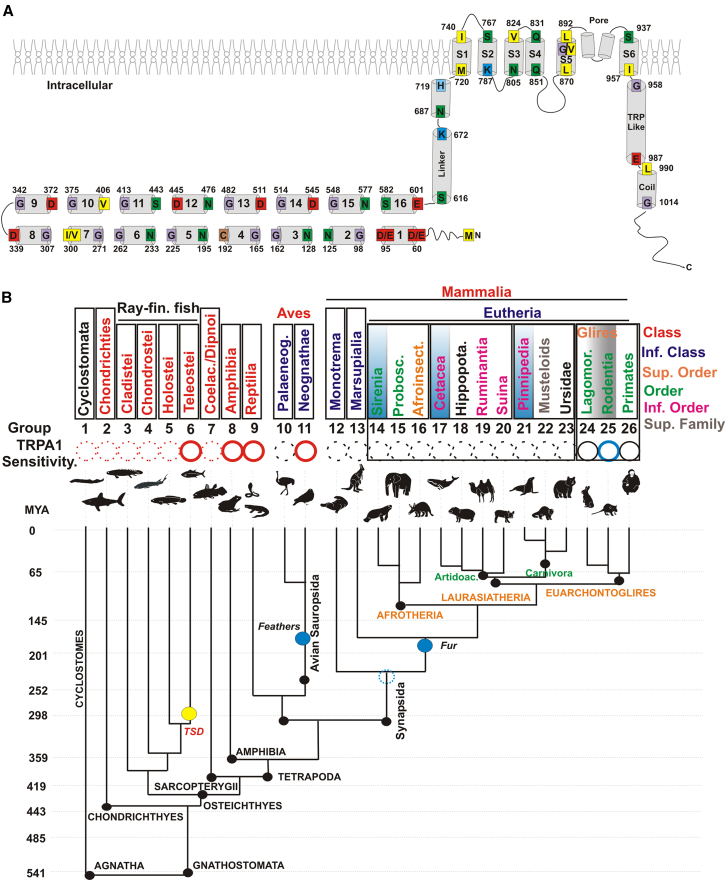
Representative TRPA1 Structure and Phylogenetic Diagram of Related Taxa (A) Schematic of the secondary structure of human TRPA1: The 16 ankyrin repeat domains (ARD) at the amino terminus are numbered, followed by the linkers, the six transmembrane domains (S1 to S6), the TRP like domain, and the coiled coil domain at the carboxy terminus. (B) Related taxa (classes or superfamily) of vertebrates with similar TRPA1 “thermosensation” as determined by electrophysiological data obtained from the literature are color coded (heat, red; cold, blue; temperature insensitive, black; and unknown, dashed circles). Extant species of interest shown separated (see Materials and Methods) into 26 groups corresponding to the colored taxonomic clades indicated on the right. Evolutionary years (millions), dividing different eras, are approximated and are indicated on the left. Teleost-specific duplication [TSD] is indicated in yellow. Blue circles denote the age of the oldest preserved fossils with fur or feathers with insulation capacity, likely related to the advent of homeothermy (groups 10 to 26). Groups 1 to 9 are ectotherms. Groups 14 to 23 (square) are analyzed in Figure 8 and contain several specific examples of marine mammals, while groups 24 to 26 (square) are analyzed in Figure 7 and contain taxonomic groups with cold-activated TRPA1 (group 25, some rodents) and temperature-insensitive TRPA1 (group 24, Sciuromorpha and lagomorphs; group 26, primates).

Thus, we compared in distinct extant species the molecular structure of TRPA1 as related to the temperature range of the channel as identified previously by electrophysiology. Of note, mechanical and chemical stimuli were not considered in our analysis. The molecular structure of TRPA1 consists of six transmembrane α-helices (S1–S6), a re-entrant pore loop between S5 and S6, and variable numbers (14–18) of amino terminal ankyrin repeat domains (ARD).^5^^,^^58^^,^^61^ For instance, the Trpa1 of *Xenopus* and humans contains 16 ARDs (Figure 6A). A cluster of ARDs located at the amino terminal end of the protein is thought to be the “sensor domain” of TRPA1.^5^^,^^6^^,^^61^^,^^63^

To determine how changes in the thermal sensitivity of TRPA1 mapped onto evolutionary lineages, we compared extant organisms grouped into 26 clades (taxonomic classes to infraorders) as referenced by the Integrated Taxonomic Information System (ITIS) (https://www.itis.gov/) (Figure 6B; Table S2). These organisms were grouped into clades based on their shared evolutionary history and genetic similarities, providing a structural framework for analyzing thermal sensitivity trends. We used the electrophysiological literature to identify the thermal sensitivity range of TRPA1 in species across these clades. Our analysis focused exclusively on Chordata (organisms with an internal cartilaginous or bony skeleton) as the mechanisms to change skin color differ from those organisms in Protochordata. To the best of our knowledge, the temperature sensitivity of Trpa1 in lamprey (Cyclostomata; Group 1), sharks (Chondrichthyes; Group 2), and most ray-finned fish (Groups 3 to 5) has not yet been investigated by electrophysiology. Trpa1 electrophysiology was performed in Teleostei (Group 6), which possess two genes due to a specific genome duplication (TSD, Teleost specific duplication) (Figure 6B). In several Teleostei organisms (zebrafish, medaka, and takifugu), at least one of the two Trpa1 homologues is activated by heat.^55^^,^^64^^,^^65^ Trpa1 is also heat-activated in amphibians (Group 8), reptiles (Group 9), and birds (Aves; Group 11)^5^^,^^7^^,^^57^^,^^66^ (Figure 6B). There is no physiological data for TRPA1 in organisms from Group 10, which includes flightless birds (Aves, infraclass Palaeognathae), while chicken TRPA1 (Group 11; Aves, Neognathae including most of flying birds) has an activation threshold of 39.4°C, a temperature below their typical body temperature (40°C–42°C)^57^ (Figure 6B). In the mammalian taxonomic class (Groups 12 to 26), TRPA1 electrophysiological data are available for placental organisms (Eutherian), revealing a thermal shift of TRPA1 with respect to ectotherms (Figure 6B), but are lacking for monotremes (Group 12) and marsupials (Group 13). Thus, the temperature dependency of TRPA1 appears correlated with its function in thermoregulation over evolution, acting as a heat sensor in ectotherms and shifting with the advent of fur and feathers.

**Figure 7:**
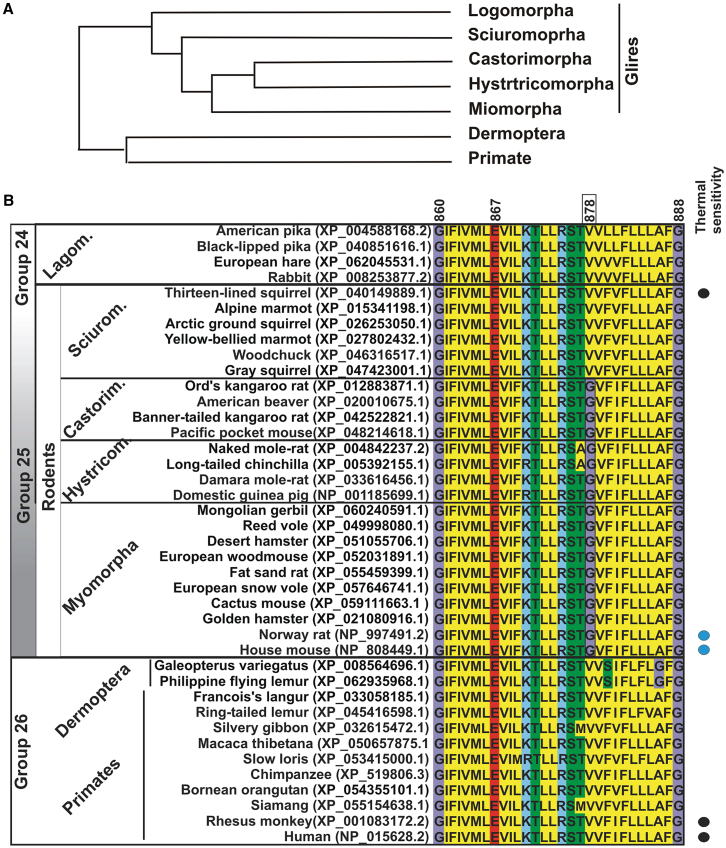
Phylogenetic divergence and alignment of the S5 Region of Glires and Primates (groups 23 to 26) as indicated in Figure 6 (A) Phylogenetic analysis (not to scale) from all major rodent clades modified from Blanga-Kanfi and co-workers^43^ based on six nuclear genes. (B) S5 region alignment containing the glycine residue (G878) present in several rodents (Group 25: Myomorphas, Hystricomorphas, and Castorimorphas) that is essential for cold sensitivity.^9^ The substitution of this residue with valine (V) in primates (Group 26) and lagomorphs (Group 24: rabbits, hares, and pikas) correlates with, where data are available, instances of thermal differences in TRPA1 sensitivity. Substituting human V878 with G878, however, does not restore cold sensitivity. Note that TRPA1 in the rodent squirrel (Sciuromorpha) is temperature insensitive and contains V at amino acid 878 instead of G.^67^ Demonstrated electrophysiological data for TRPA1 thermal sensitivity (cold, blue; insensitive, black) are indicated on the right.

Notably, the supra-order Euarchontoglires, which contains rodents and primates (Groups 24 to 26), exhibited a shift in TRPA1 thermal sensitivity to cold or insensitive. In Glires (groups 24 and 25) that contain the rodents (order Rodentia; Group 25), the electrophysiological data clearly indicates cold temperatures activate Trpa1 in mice, rats, and guinea pigs, but not in squirrels.^5^^,^^9^^,^^20^^,^^67^ Indeed, in squirrels, as in primates, TRPA1 appears to be cold insensitive.^67^ Interesting, the squirrel lineage diverged early during rodent evolution, immediately after the divergence of Lagomorphs^43^ (order Lagomorpha includes rabbits, hares, and pikas) (Figure 7A). Here, we also found a correlation with the presence of a specific amino acid located within the S5 transmembrane domain that is essential for TRPA1 cold thermal sensitivity, Valine 878.^9^ V878 is present in all Primates and Dermoptera (group 26), lagomorphs, as well as in the rodent lineage that generated squirrels (suborder Sciuromorpha) (Figure 7B). A substitution at this residue (878 V to Glycine) occurred in the rodent lineage that generated Myomorphs (mice and rats), Castorimorphs (castors), and Hystorimorphs (mole rats) (Figure 7B), indicating a correlation between this amino acid and the available TRPA1 thermal sensitivity data in Glires and primates. Note, however, that while this residue is essential for cold sensitivity it is not sufficient on its own to explain the thermal shift observed in mammals, in that converting V878 to G878 in human TRPA1 does not induce cold sensitivity.^9^

Finally, we analyzed the two additional Eutherian supra-orders, Laurasiatheria and Afrotheria (Group 14 to 23) (Figure 6B). While we were unable to find TRPA1 electrophysiological data for these two supra-orders, they were particularly interesting as they contain extant species that adapted to aquatic environments at different evolutionary times and with distinct thermoregulatory mechanisms.

While a single residue at the pore, such as G878, is essential for mouse TRPA1 cold sensitivity, several studies suggest the domain responsible for thermosensing is in the ARD region. First, the three-dimensional structure of human TRPA1 obtained by single-particle electron cryomicroscopy shows a close proximity between the ARD and helix-turn-helix motifs that allow the ARD to transmit information to the pore.^58^ Second, an unbiased random mutational analysis performed for mouse TRPA1 shows that the alteration of three amino acids in ARD6 switches thermal sensitivity from cold to heat.^63^ Moreover, two snakes with significantly different thermal activation temperatures for Trpa1 (28°C vs. 38°C) experience thermal shifts when the amino terminal regions of their proteins are interchanged.^6^ Finally, when the thermally insensitive human TRPA1 is engineered to contain the first 10 ankyrin repeats from a rattlesnake, the protein gains heat sensitivity.^6^

**Figure 8:**
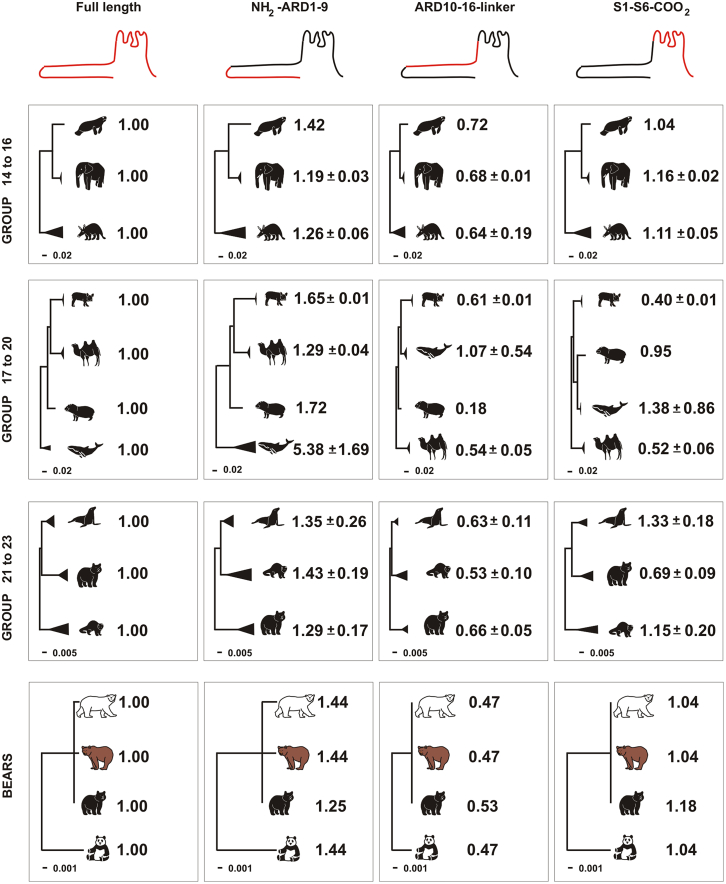
Comparative phylogenetic analysis of TRPA1 domains for three marine mammals with terrestrial relatives Molecular phylogenetic analysis by the maximum-likelihood method based on the JTT matrix model was performed with the amino acid sequences of species shown in Table S2. The trees with the highest log-likelihood are shown to scale. Note the scale changes between the more recent evolutionary event containing bears (polar bear; brown bear; black bear and panda) and seals (Pinnipeds; groups 21–23) with respect to the analysis with manatees (Sirenia; groups 14 to 16) and whales (Cetacea; groups 17 to 20). The full length TRPA1 sequence, the region containing the sensor with the ankyrin repeat domain (ARD) 1 to 9, ARDs 10 to 16, and the transmembrane domains (S1 to S6) were used for the analysis. Numbers in the right (mean ± SD) indicate the phylogenetic distance relative to that for full-length TRPA1. Lack of standard deviation indicates only one species with TRPA1 data available. Evolutionary analyses were conducted in MEGA X.^68^

We performed a mutational analysis for four examples of mammalian lineages that transitioned from terrestrial or freshwater aquatic environments to the sea, in that these lineages provide examples where thermoregulation evolved to rely on cardiovascular adjustments rather than skin pigmentation. In marine mammals, blood flow to the skin is often reduced during diving to conserve oxygen for vital organs, which also helps retain body heat. Conversely, increased blood flow to the skin at the surface or in warmer waters aids in heat dissipation.^35^ Additionally, the existence of a thick blubber layer for thermal insulation generally correlates with earlier aquatic adaptation, while the existence of fur is characteristic of more recently adapted organisms, as hair can increase drag in aquatic environments.^35^^,^^36^^,^^39^ We analyzed the mutation rates of TRPA1 structures associated with thermoregulatory physiology. Using the maximum-likelihood method based on the JTT (Jones-Taylor-Thornton) matrix model, which is an empirical substitution model commonly used in evolutionary studies to estimate amino acid replacement rates, we compared three regions from the full-length TRPA1 (TRPA1 full length): 1) the amino-terminal region that contains nine ARDs (NH_2_-ARD 1–9) and likely where thermal sensitivity resides; 2) a middle domain with ARD10-16, plus two linker domains (ARD10-16-linker); and 3) the transmembrane regions, where the pore is localized, and carboxy-end (S1-S6-COOH) regions (Figures 6A and 8 scheme). We hypothesized that the rate of amino acid change in the sensory region (ARD1-9; Figure 6A) of TRPA1 would be highest in lineages that transitioned earlier to the sea. We also predicted, in contrast, that mutation rates for the transmembrane S1-S6 domain containing the pore for cation influx would remain low in all four lineages, as this domain is linked to the evolutionarily conserved selectivity of ion influx for non-selective cation channels.^69^ Indeed, the mutational rates in the pore region were low (Rate close to 1) (Figures 6A and 8).

From the numerous independent evolutionary events in which mammals adapted to aquatic environments, we focused on four groups of extant species that exhibit distinct skin characteristics:

1) Sirenians (manatees and dugongs), also known as sea cows, possess thick, wrinkled skin covered with short, fine sensory hairs. Their blubber layer is notably thinner as compared to other marine mammals, likely due to the warmer aquatic niche that they inhabit (20°C–30°C).^39^ Having adapted to an aquatic environment approximately 50 million years ago (MYA), manatees share an evolutionary relationship with two clades of extant terrestrial species: elephants (order *Proboscidea*) and Afroinsectiphilia (African insectivores), which include golden moles, aardvarks, and Cape elephant shrews [(Groups 14–16) (Figures 6B, 8, and Table S2)]; 2) Cetaceans (whales, dolphins, and porpoises) that are entirely adapted to a cold aquatic lifestyle, with a smooth skin (hairless) and a thick layer of underlying blubber. This blubber provides insulation, buoyancy, and energy storage and is highly vascularized.^38^ Cetaceans became aquatic from a common terrestrial ancestor of hippos and whales (Whipomorpha) at approximately the same evolutionary time as Sirenians (50 MYA), diverging from a lineage that includes extant terrestrial species such as peccaries (Suina) and ruminants (Ruminantia) [(Group 17 to 20) (Figures 6B, 8, and Table S2)]; 3) Pinnipeds (seals, sea lions, and walruses), such as Cetaceans, have a skin with a thick blubber layer, but differ in that their integument is covered with short, dense fur, likely reflecting a recent adaptation to an aquatic environment (20 MYA).^36^^,^^40^ Pinnipeds can additionally regulate body temperature through spending time on land, mainly during molting.^70^^,^^71^ Pinnipeds are carnivores that are evolutionarily related to minks, otters, and ferrets (Musteloidea), and bears (Ursidae) [(Group 21 to 23) (Figures 6B, 8, and Table S2)]; Finally, 4) Polar bears, considered marine mammals due to their reliance on the marine environment for hunting. Polar bears lack a typical blubber layer, possessing a dense fat layer in the skin with a distinctive composition, alongside the ability to secrete fur sebum with anti-icing properties.^42^ Additionally, polar bears and sea otters are insulated by air trapped in their thick fur, containing millions of hairs per square inch.^72^^,^^73^ We compared TRPA1 from polar bears with their terrestrial bear relatives (Brown bear, black bear, and giant panda) [(Group 23) (Figures 6B, 8, and Table S2)].

We analyzed the phylogenetic distance between these four groups and compared the full-length TRPA1 protein against the three regions of interest (expressed as a ratio: region of interest/full length) across representative members of each group. For all four groups, the aquatic and terrestrial members of the group showed similar low mutation rates in the S1-S6 and ARD10-16 linkers (Figure 8). In contrast, those lineages that became aquatic early in evolution, the manatees and whales, appeared to show higher mutation rates in the amino acid sequence of their ARD1-9 in comparison to their terrestrial relatives [ratios: Group 14 (1.42; manatees) vs. Group 15 (1.19; elephants); Group 17 (5.38; whales) vs. Group 18 (1.72; Hippo); Figure 8)]. In contrast, for seals/sea lions and polar bears, which evolved more recently, the mutational rates in the ARD1-9 region were not higher, when expressed as a ratio to the mutational rates of the full-length TRPA1, to those obtained for their corresponding relatives [ratio: Group 21 (1.35; seals/sea lions) vs. Group 22 (1.43; Musteloidea) and 1.44 for polar, panda and brown bears)].

Together, these results indicate that the thermosensitivity of TRPA1 changed over the course of evolution, coinciding with specific evolutionary events. The protein shifted from functioning as a heat sensor in fish, amphibians, and reptiles to likely losing its noxious heat function during the evolution of thermoregulation, which is associated with the emergence of feathers and fur in the integument of homeotherms. In rodents (e.g. mice and rats), TRPA1 functions as a cold sensor. However, TRPA1 in the squirrel lineage (also rodents) and primates appears to be thermally insensitive. Finally, we observed an increased mutational rate in the sensory region of TRPA1 in early adapted marine mammals (e.g. manatees and whales) compared to their terrestrial relatives.

## Discussion

Changing skin color is a crucial physiological process that aids in the thermoregulation of ectotherms. We demonstrate that TRPA1 functions as a heat sensor, regulating temperature-induced changes in skin pigmentation in *Xenopus laevis*. These data provide support for the idea that TRPA1 thermal sensitivity correlates with integumentary traits. TRPA1 serves as a heat sensor in ectotherms with uncovered integuments (e.g. fish, amphibians, and reptiles), and shifted its thermosensitivity likely during the evolution of thermoregulation. Our evolutionary analysis, although based on incomplete electrophysiological data, raises the possibility that TRPA1 became thermally insensitive in the Euarchontoglires lineage and subsequently evolved cold sensitivity in several lineages of rodents. Finally, our analysis suggests that the thermosensor region of TRPA1 of marine mammals experienced reduced selection pressure in lineages that adapted early to marine environments (e.g. manatees and whales) with thick, sparsely haired skin, but showed reduced mutagenesis in organisms covered with dense hair that adapted more recently (e.g. sea lions and polar bears). Skin is the largest “organ” of an organism’s body with essential thermoregulatory functions. We propose that TRPA1 functions in skin physiology and its thermal sensitivity evolved hand in hand during evolution.

### Trpa1 regulates melanosome dispersion in amphibians, impacting pigmentation as a thermoregulatory mechanism

In this study, we observed that *Xenopus laevis* tadpoles darken their skin rapidly in response to moving to an environment with an ecologically relevant warm temperature (32°C). This darkening occurs via melanosome dispersion within melanophores and is both reversible and regulated in a cell autonomous manner. We propose that Trpa1 acts in melanophores to mediate the dispersion of melanosomes. In support, we find mRNA for Trpa1 in tadpole tails and melanophore MEX cells. Further, Trpa1 is co-expressed with Trpv1 by skin melanophores. While melanophores co-express Trpa1 and Trpv family members, pharmacological studies with specific agonists and antagonists, combined with molecular knockdown of Trpa1, argue strongly that Trpa1 induces the skin darkening response to heat. In support of Trpv1 playing no role, the activation threshold for *X. laevis* Trpv1 is approximately 40°C, and calcium influx through the channel is undetectable at 34°C for *X. laevis* Trpv1-transfected HeLa cells,^8^ both temperatures higher than our thermal-testing paradigm.

In *X. laevis*, we find two *trpa1* genes: *trpa1.L* and *trpa1.S*, though we find that only the L form appears to be expressed in *Xenopus* melanophores, as suggested by previous work showing that while *trpa1.L* is expressed in brain, spinal cord, skin, and peripheral neurons, *trpa1.S* mRNA is restricted to the brain.^8^ *Xenopus* Trpa1 appears sensitive to temperatures in a complementary range to that of Trpm8, with the former mediating skin darkening in response to heat, and the latter skin lightening in response to cold conditions.^15^ Interestingly, the activation of Trpm8 also impacts tadpole locomotor performance, while anuran tadpoles across several species exhibit Trpa1-mediated heat avoidance behavior.^74^ Thus, pigmentation and thermal behavioral responses appear connected, with hot and cold temperatures sensed by Trpa1 and Trpm8, respectively. Note that the temperature paradigm we chose was based on the thermal characteristics of the *Xenopus laevis* niche. Although Trpa1 functions as a heat sensor in ectotherms, small differences in Trpa1 thermal sensitivity exist even between closely related species. For example, *Xenopus tropicalis*, found in western tropical Africa, has a higher Trpa1 activation threshold than *Xenopus laevis*, which is found in colder southern Africa.^8^^,^^44^^,^^75^ Presumably, these differences reflect distinct needs for thermal behavior responses and/or thermoregulatory skin pigmentation in different environmental niches.

### The evolutionary shift in TRPA1 thermal sensitivity is closely linked to the thermoregulatory role of melanin in pigmentary physiology

TRP channel expression is highly conserved between ectothermic melanophores and mammalian melanocytes. Indeed, our results show that *Xenopus* melanophores express multiple and similar TRP channels, including Trpm8,^15^ Trpa1, Trpv1, Trpv2, and Trpv4, to those detected in mammalian melanocytes and melanoma cells.^54^^,^^76^ The conservation of Trpa1 expression between melanophores and melanocytes, alongside melanin’s role in ectothermic thermoregulation^1^^,^^2^^,^^27^ and evolutionary changes in TRPA1 thermosensitivity,^4^^,^^19^^,^^61^ suggest a connection between the emergence of endothermy and pigmentary physiology. Our systematic analysis of TRPA1 thermosensitivity, based on electrophysiological data from the literature, underlines the function of Trpa1 as a heat sensor in ectotherms and its modification or shift in endotherms. While we focused on Chordata, invertebrate data argue that Trpa1 originally acted as a heat sensor [Reviewed by^4^^,^^20^]. Our findings suggest that TRPA1 thermal sensitivity evolved alongside the advent of endothermy, correlating with changes in the localization and function of pigmented cells and melanin in skin physiology. Indeed, alterations in the location of melanin pigment-containing melanosomes over evolution are associated with changes in thermoregulatory mechanisms. For instance, in amphibians, melanin-filled melanosomes are largely distributed to the pigmented cells of internal organs and not integumental melanophores,^28^^,^^77^ though melanin’s thermoregulatory function is likely restricted to the latter cell population. Melanin-containing pigment cells became distributed equally between the skin and internal organs of reptiles,^78^ potentially enhancing the integumentary role of melanin in thermoregulation as animals transitioned to land. With the evolution of endothermy, integumentary melanosomes became dominant, and pigmented cells in the integument acquired a secretory role (birds and mammals),^78^ coinciding with the evolution of feathers or hair for thermoregulation. Our analysis of TRPA1 thermal sensitivity in Chordata suggests a similar switch over evolution that coincided with changes in the involvement of the integument in thermoregulation.

The timing of the shift of TRPA1 from a heat sensor to a thermally insensitive or cold sensor during evolution remains unknown. Our findings, however, do suggest that TRPA1 was already insensitive to temperature at the base of Euarchontoglires (see later in discussion). In humans, UV light rather than temperature activates TRPA1 in melanocytes, inducing melanin synthesis.^23^^,^^24^^,^^25^ The mechanism involved remains unknown and additional studies are necessary to determine whether the UV activation of human TRPA1 is direct or indirect. Is the UV-induced activation of TRPA1 enhanced in hominid skin as an adaptation of the “hairless integument” for UV protection? Research answering that question could reveal whether this adaptation emerged/increased during the savanna period in response to hair loss or if it was conserved in hairy mammals.^30^^,^^31^ This information is particularly relevant in that cutaneous-specific TRPA1 knockout mice exhibit reduced sensitivity to mechanical stimuli and temperature.^11^

### Cold thermal sensitivity detected in certain rodent lineages correlates with the phylogeny of Euarchontoglires with the substitution of an essential single amino acid (V→G878)

Interestingly, our evolutionary analyses suggest that TRPA1 was thermally insensitive in early ancestors of Euarchontoglires, but that a mutation in amino acid 878 from valine to glycine was in part responsible for conferring cold sensitivity in certain rodents. The cold thermosensing of mouse and rat TRPA1 relates to this amino acid being a glycine, in that a mouse TRPA1 version where G878 is mutated to V878 is thermally insensitive.^9^ In squirrels, however, temperatures as low as 10°C fail to activate the channel.^67^ Our evolutionary analysis of more than 50 extant rodent species reveals that the squirrel lineage retains a V878. While G878 is essential for cold sensitivity in mice and rats, this variant is not sufficient by itself to determine cold sensitivity, as human TRPA1 does not regain cold thermal properties when V878 is switched to G878.^9^ Although an independent amino acid substitution in the different lineages cannot be excluded, the evolutionary relationships within Euarchontoglires strongly support the possibility that V878 was the ancestral state in this lineage. Thus, TRPA1 was likely thermally insensitive at the base of the Euarchontoglires lineage, as V878 remains in Sciuromorphs, Lagomorphs, and primates, but mutated to G878 specifically in Myomorphs (mice and rats), Hystricomorphs (mole rats), and Castorimorphs (beavers). A phylogenetic analysis of Rodentia, based on six nuclear genes from 41 rodent species, supports this hypothesis—showing that squirrels (Sciuromorphs) diverged early, after lagomorphs and primates, while all other rodent clades emerged later from a common ancestor.^43^ Additional electrophysiological data from other species of Glires are necessary to support this hypothesis.

### Reduced TRPA1 selective pressure in marine mammals correlates with the loss of fur

We find a higher mutation rate in the thermosensory region of TRPA1 that correlates with the adaptation of individual marine mammals to aquatic environments and the reorganization of their integumentary structure. Marine mammals underwent distinct evolutionary adaptations, ranging from morphological (e.g., streamlined body shape, paddle-like limbs for enhanced mobility) to physiological changes (e.g. echolocation, thermoregulation). This is especially true for skin physiology, where differences, such as the presence of fur or a thick blubber layer, vary depending on when certain species adapted to a marine environment. Over evolution, mammals re-entered the marine realm at least seven separate times. Five of these lineages possess extant species.^37^ Here, we analyzed the TRPA1 of four of them. The fifth lineage corresponds to marine otters that adapted to the sea environment 5 MYA. Marine otters are carnivores of the Mustelidae family (Group 22), closely related to polar bears (Group 23; Ursidae), sharing several skin structures. Therefore, we did not analyze them as an independent group.

Our data show that the mutation rate in the thermosensory region of TRPA1 is especially elevated in the two earliest adapted (50 MYA) marine mammal lineages, the Sirenians and Cetaceans. These lineages lack typical mammalian fur and instead possess a thick skin with a substantial blubber layer used for thermal regulation.^38^^,^^39^ Interestingly, the smooth, drag-reducing skin of cetaceans, which is keratinized, lubricated, and hairless, resulted from specific episodes of gene loss.^38^ In agreement, we detected a higher mutational rate of cetacean TRPA1, suggesting low selection pressure. In contrast, pinnipeds, which evolved around 20 MYA, feature a fur-covered integument with a thinner blubber layer than whales.^40^ The blubber of cetaceans and pinnipeds is highly vascularized, in part to regulate thermal dissipation.^35^ Note that pinnipeds also thermoregulate by spending time on land.^70^^,^^71^ Pinnipeds, as well as polar bears, show a mutation rate in the TRPA1-thermosensitive region that appears to be similar to that of their terrestrial relatives. While the integument of polar bears is highly pigmented (melanin), solar energy does not reach the skin epithelium of the thick dorsal fur, and only partially does in thinner fur regions.^73^ Thus, thermoregulation in polar bears appears to rely mainly on fur characteristics^42^ and the integumentary adipocyte layer, and not melanisation. Thus, in general, our data indicate a correlation with less pressure selection on TRPA1 thermosensing in early adapted marine mammals that depend less on skin-mediated thermoregulation than the more recently adapted marine lineages.

TRPA1 acts as a heat sensor in amphibians, and this thermal sensitivity plays a crucial role in melanin dispersion within the integumentary melanophores of ectotherms. In organisms with uncovered integument (ectotherms), thermoregulation is partially mediated by the dispersion and aggregation of melanosomes. Interestingly, the role of integumentary melanin evolved alongside the transition to endothermy, coinciding with shifts in TRPA1 thermal sensitivity. We propose that TRPA1 functions in skin physiology and its thermal sensitivity co-evolved throughout vertebrate evolution.

### Limitations of the study

This article examines Trpa1’s role in melanosome distribution in ectotherms using *Xenopus laevis* as a model organism. While melanin dispersion contributes to thermoregulation in many ectothermic species, its impact is generally more substantial in terrestrial animals. Nonetheless, *Xenopus laevis*, though aquatic, exhibits a pronounced melanism response in which Trpa1 plays a key role. Given the observed involvement of Trpa1 in this response in an ectotherm, further analysis across species is needed. The second part of our study investigates TRPA1’s thermal sensitivity across extant chordates using published electrophysiological data. Our findings support an evolutionary transition of TRPA1 from a heat-activated channel to one responsive to cold or thermally insensitive. Although we primarily relied on studies examining TRPA1 electrophysiology under similar conditions across multiple species, recent work from Vlachova’s lab^79^^,^^80^^,^^81^ suggests that human and mouse TRPA1 may act as a bidirectional thermosensor, capable of detecting both heat and cold. A major limitation of our approach is the scarcity of electrophysiological data in species outside the Glires clade and the lack of comprehensive evolutionary analyses of functional TRPA1 across lineages. These gaps hinder efforts to pinpoint the timeline and mechanisms underlying TRPA1’s thermal transition. Further electrophysiological studies in additional taxa are essential to validate and expand on the correlations observed.

## Resource availability

### Lead contact

Further information and requests for resources and reagents should be directed to and will be fulfilled by the lead contact Sarah McFarlane (smcfarla@ucalgary.ca)

### Materials availability

This study did not generate new unique reagents. Antibodies, reagents, and the cell line use were obtained from commercial or other sources as outlined in the key resources table.

### Data and code availability

• Data have been deposited at general-purpose repository Dryad and are publicly available as of the date of publication at https://doi.org/10.5061/dryad.wpzgmsc0t.
• This article does not report original code.
• Any additional information required to reanalyze the data reported in this article is available from the lead contact upon request.

## Acknowledgments

This work was supported by an operating grant from the Natural Sciences and Engineering Research Council of Canada (10.13039/501100000038NSERC) to SM. We thank Ms. Carrie Hehr for excellent technical assistance.

## Author contributions

GEB and SM designed the experiments. GEB and NH performed the experiments and analyzed the data. GEB and SM supervised the study and wrote the article.

## Declaration of interests

The authors declare no competing interests.

## STAR★Methods

### Key resources table

**Table:** 

REAGENT or RESOURCE	SOURCE	IDENTIFIER
**Antibodies**		

Anti-crocodile Trpv1 (rabbit polyclonal)	Thermo-Fisher, Scientific	Cat #: OST00058W
Anti human TRPA1 (rabbit polyclonal)	Biorbyt	Cat #: orb575403
Anti human Tyrp-1 (rabbit polyclonal)	Thermo-Fisher Scientific	Cat #: PA5-81909; RRID: AB_2789070
Secondary antibody Alexa Fluor 488	Invitrogen	Cat #: A11008; RRID: AB_143165

**Bacterial and virus strains**		

DH5a Chemically Competent E. coli	Invitrogen	Cat #:18258012
TOP10 Chemically Competent E. coli	Thermo-Fisher, Scientific	Cat #: C404010

**Experimental models: Cell lines**		

Xenopus Melanophore cell line (MEX)	Dr Rodionov, V.I.^47^	
Xenopus laevis J. strain	Xenopus1.Corp	

**Chemicals, peptides, and recombinant proteins**		

A-967079	Millipore Sigma	Cat #: SML0085
Allyl isothiocyanate (AITC)	Millipore Sigma	Cat #: W203408
Capsaicin	Millipore Sigma	Cat #: M2028
Capsazepine	Abcam	Cat #: Ab120025
GSK2193874	Tocris Bioscience	Cat # 5106
GSK1016790A	Millipore Sigma	Cat #: G0798
Piperine,	Abcam	Cat #: Ab142933
1-phenyl-2- thiourea (PTU)	Sigma Aldrich	Cat #: P7629
SuperScriptTM IV reverse transcriptase	Invitrogen	Cat #: 18090050
TOPO™ TA Cloning™	Thermo-Fisher Scientific	Cat #: K4575J10
Lipofectamine 2000	Thermo-Fisher Scientific	Cat #:11668027
Hoechst 33258	Millipore Sigma	Cat #: 861405

**Oligonucleotides**		

See Table S1		

**Software and algorithms**		

GraphPad Prism Version 10.1.1.	GraphPad	
CorelDraw Version 10.0	Corel Draw	
Adobe Photoshop PS Version 23.4	Adobe Photoshop	
ZEN Microscopy Software	Zeiss Microscopy	
AxoVision Microscopy Software	Zeiss Microscopy	
MEGA X Version 11.0.13	Dr Kumar S.^78^	Public domain
ImageJ 1.54g	NIH. USA	Public domain

### Experimental model and study participant details

#### Embryos

The Animal Care and Use Committee, University of Calgary, approved procedures involving frogs and embryos (AC21-0148; signed by Dr. Derrick Rancourt). Embryos were obtained by induced egg production from chorionic gonadotrophin (Intervet Canada Ltd.) injected females and *in vitro* fertilization according to the standard procedures (see protocols at Xenbase (http://www.xenbase.org). Embryos were maintained at 16°C in Marc’s modified Ringer’s (MMR) solution (100 mM NaCl, 2 mM KC1, 2 mM CaCl_2_,1 mM MgCl_2_, 5 mM HEPES pH 7.4) until stage 43/44 (approximately 1 week) and staged according to Nieuwkoop and Faber on Xenbase (www.xenbase.org). Of note, embryos at this stage cannot be sexed. The embryos were reared under light cycles of 12 h ON/12 h OFF (light = 1000 lux or approximately 1.5 × 10–4W/cm^2^) on a white background.

#### Cells

To test the pigmentation response to temperature we employed the melanophore (MEX) cell line originally generated from stage 35 *Xenopus laevis* embryos.^47^ Cells were maintained in growth medium (65% Leibovitz’s L15 medium with 30% added water and supplemented with 5% fetal bovine serum (Invitrogen) without antibiotics). Although the cells have not been formally authenticated, several traits confirm their identity as a *Xenopus laevis* melanophore cell line: presence of melanin, ability to grow in medium diluted in water (60/35%), proliferation at room temperature without CO_2_ supplementation, and PCR amplification of multiple genes using *X. laevis*-specific primers. Hoechst 33258 staining (1 μg/mL) suggests the cell line is free from mycoplasma contamination. To mimic the *in vivo* conditions of tadpoles, cells were maintained in medium without phenol red to maximize light penetration. For the pigmentation heat response, cultures at 16°C were switched to 32°C. During the warming paradigm, light from above (1000 lux) was maintained, identical for tadpoles and MEX cells. Cells were fixed with 4% paraformaldehyde and stained with DAPI (1 μg/μL) before imaging.

### Method details

#### Pigmentation index

We quantified changes in skin pigmentation by measuring skin indices as described previously.^82^ Briefly, pictures of the dorsal head of tadpoles were taken using a stereoscope (Stemi SV11; Carl Zeiss Canada, Ltd., Toronto, Canada) and a camera (Zeiss; Axiocam HRC), with identical conditions of light, exposure time and diaphragm aperture. The pictures were converted to binary white/black images using NIH ImageJ (U. S. National Institutes of Health, Bethesda, MD). Examples of pictures before conversion to binary images are show in Figure 1B.

#### MEX cell dispersion

Dispersion/aggregation of melanosomes on MEX cells melanophores was determined after different temperature treatment on fixed cells. Pictures of cells stained with DAPI (1 μg/ml) and bright field of the same area were used for quantification. Example of pictures with dispersed and aggregated melanosomes are shown in Figure 2A.

#### Warm treatment

For warm treatment experiments, embryos or MEX cells were set in 35 mm dishes with 4 mL of MMR or growth medium, respectively, at 16°C before switching to a 32°C incubator for the indicated times. Tadpoles and cells were reared under light cycles of 12 h ON/12 h OFF (light = 1000 lux or approximately 1.5 × 10–4W/cm^2^) on a white background. A 32°C non-noxious warm temperature was chosen based on the average temperatures recorded over the last 30 years for three national parks in southern Africa (Etosha/Namibia; Kriger/South Africa and Hawange/Zimbawe) obtained from the Meteoblue website (https://www.meteoblue.com/en/weather/historyclimate/).

#### Effect of TRP agonists and antagonists

TRP agonists were added to embryos maintained at 16°C, while antagonists were added at 16°C before switching embryos to 32°C. The maximum dose tested varied depending on either solubility in aqueous solution or drug toxicity. The agonists and antagonists used are organic compounds with low solubility in aqueous solutions, therefore were initially dissolved in dimethyl sulfoxide (DMSO) before dilution (1/1000) in MMR or growth medium. For example, the highest piperine dose tested was 100 μM (diluted from a 100 mM DMSO stock solution) as its maximum solubility in water is 140 μM. Toxicity causing death of tadpoles was identified by stasis after 1 h after switching to a drug-free condition. Note that in general, toxicity and death were following by skin lightening.

#### Identification/expression of TRP channels

The screening and identification of *Xenopus laevis trpv* and *trpa* members was described recently.^15^ All *Xenopus* TRP channel genes are available on Xenbase database (www.Xenbase.org). The expression of *trp* mRNAs in whole stage 43/44 embryos and melanophores was assessed by RT-PCR. Total RNA was obtained from whole embryos, surgical isolated tails, and MEX cells using TRIzol (Invitrogen) according to the manufacturer’s protocol. Single-strand cDNA was produced from RNA samples (5 μg) by priming with oligo(dT) primers using C according to the manufacturer’s instructions. All PCR amplifications were carried out in a total volume of 20 μL with 1 μL of cDNA, 2 μL of primers, 7 μL of water and 10 μL of 2X PCR master mix (Thermo Scientific, IL). PCR amplifications were carried out between 40 and 45 cycles and with an annealing temperature of 55°C. PCR products obtained from cDNA were cloned into TOPO-pCRII (Invitrogen) vectors and sequenced to confirm identity. Specific primers were designed to amplify both homologous variants (on L and S chromosomes) of the different Trp channels (Table S1). The sequence of the different *trp* channels can be obtained from GenBank-NCBI database with the gene name accession numbers provided in Figure 3A.

#### Immunohistochemistry for Trpv1 and Trpa1

Immunohistochemistry against Trpv1 and Trpa1 was performed as described recently^15^ using the following antibodies: anti-crocodile Trpv1 (rabbit polyclonal; 1/200 dilution; Thermo-Fisher, Scientific; #OST00058W) and anti-human TRPA1 (rabbit polyclonal; 1/200 dilution; Novus Biologicals; NB110-4076SS). The identification of skin melanophores was performed with a rabbit polyclonal antibody against human TYRP-1 (1:200 dilution; Thermo-Fisher Scientific, IL; PA5-81909)], a specific enzyme involved in melanin synthesis. Since antibodies against Trp channels and the melanophore marker (Tyrp-1) were all generated in rabbit, the analysis of co-expression was performed in consecutive 12 μm transverse frozen cryostat sections obtained from stage 43/44 embryos. Following detection with primary antibodies, slides were treated with a secondary antibody (1:1000 dilution of Alexa Fluor 488) and DAPI (1 μg/μL) to stain nuclei.

#### Grouping in clades

Vertebrates were divided into 26 groups based on taxon clades [(Integrated Taxonomic Information System (ITIS) (https://www.itis.gov/)] with demonstrated evolutionary relationship. Group 1 contains species in the infraphylum agnathan (cyclostomes), while Groups 2 to 26 correspond to some, but not all extant gnathostomes. We initially grouped based on the taxonomic clade “Class”. The Group 2 contains the cartilaginous fish (sharks; taxonomic class, Chondrichthyes), while the ray-finned fish (Actinopterygii) were divided into 4 groups also representing taxonomic class; The Cladistei (Group 3, bichir), Chondrostei (Group 4; sturgeons and paddlefishes) Holostei (Group 5; gars and bowfins) and Teleostei (Group 6; the largest clade of bony fish with several examples of TRPA1 cloned and characterized). The following class groups are Coelacanths and Dipnoi (Group 7; Sarcopterygii, lung fish), the Amphibians (Group 8; frogs, salamanders, and caecilians), reptiles (Group 9; lizards, snakes, turtles and crocodiles), Aves (Group 10 and 11) and mammals (Group 12 to 26). The Avian Sauropods were divided into two groups representing the ‘Inferior class’ Paleognathae (Group 10), where most primitive and mainly flightless birds reside, and Neognathae (Group 11), which include almost all living flight bird species. The three mammalian infraclasses correspond to monotremes (Group 12; egg-laying mammals), marsupials (Group 13), and eutherians (Groups 14 to 26).

Groups 14 to 23 were generated to compare three marine mammals with their terrestrial relatives: i) In the superorder Afrotheria, the Sirenians (Group 14; manatees and dugongs) were compared with elephants (Group 15; Proboscidea) and African insectivores (Group 16; aardvarks and others), ii) The cetaceans (Group 17; whales) were compared to hippopotamuses (Group 18), ruminants (Group 19), and suids (Group 20; pigs), and iii) Seals and sea lions (Group 21; Pinnipeds), marine mammals, which were compared with other carnivores including minks, otters, and ferrets (Group 22; Musteloidea), and bears (Group 23; Ursidae).

The superorder Euarchontoglires (Group 24 to 26) contains most of the species where TRPA1 is well characterized, including the lagomorphs (Group 24; rabbits, hares, and pikas), rodents (Group 25; mice and rats) and primates (Group 26; human and monkeys).

#### Alignment and phylogenetic analysis

Validated sequences (NCBI numbers in Table S2) were aligned using MUSCLE (multiple sequence alignment) to build a hidden Markov model (HMM) by using a maximum-likelihood architecture construction algorithm. All phylogenetic analysis and alignments were performed by using the public domain MEGA X software (www.megasoftware.net).^68^

#### Microscopy

Images of embryos or fixed MEX cells were taken with an Axio-Cam HRc (Carl Zeiss) on the Stemi SVII stereomicroscope (Carl Zeiss). Section images from immunohistochemistry of MEX cells were processed for brightness and contrast with Adobe Photoshop.

### Quantification and statistical analysis

#### Quantification of pigmentation index

The methodology for obtaining binary images of tadpoles is detailed in the Methods section. Quantification was performed using ImageJ, a public-domain software that calculates the integral density of positive pixels over the top of the head or other specific areas, generating the 'pigmentation index’.

#### Quantification of dispersed cells

Fluorescent and bright-field overlapped images were used to quantify total cells (DAPI-positive fluorescence) and classify them based on melanosome distribution—either aggregated around the nucleus (Aggregation) or dispersed along dendritic processes (Dispersion). The percentage of dispersed cells is reported.

#### Statistics analysis and reproducibility

GraphPad Prism 10.0 software was used for statistical analysis of data and graphic preparations. Statistical analysis was ANOVA followed by Bonferroni’s test. Significance was considered at *p* < 0.05. Experiments were performed three independent times (*N* = 3) unless otherwise indicated. Analysis of dispersion of melanophores on MEX cells were performed in a minimum of 2 dishes. Random pictures of at least 6 areas were taken (*n* = 6). Since pigmentation levels vary among tadpole hatches, a representative experiment is presented. The independent experiments showed similar trends. Each experimental treatment contained a minimum of 9 tadpoles (*n* ≥ 9), which are represented in the figures as individual data points. Additionally, figures show a boxplot (25th to 75th percentile) of the mean and 95% confidence interval. Immunohistochemical analyses were performed at less three times (*N* = 3), with 4 independent tadpoles (*n* = 4) in each experiment. CorelDraw 10.0 was used to compile multipaneled figures.

## Supplemental information

Document S1. Figures S1 and Tables S1

Table S2. Excel document with the list of TRPA1 protein sequences from species grouped as indicated in Figures 6–8 with the corresponding GenBank access number
