# Supplementary material for: Evolutionary adaptations of TRPA1 thermosensitivity and skin thermoregulation in vertebrates
Source: iScience. 2025 Aug 14;28(9):113369. doi: 10.1016/j.isci.2025.113369 (PMC12446388; doi:10.1016/j.isci.2025.113369)
Supplement: Document S1. Figures S1 and Tables S1 [file mmc1.pdf]

iScience, Volume *28*

Supplemental information

Evolutionary adaptations of TRPA1 thermosensitivity and skin thermoregulation in vertebrates

Gabriel E. Bertolesi, Neda Heshami, and Sarah McFarlane

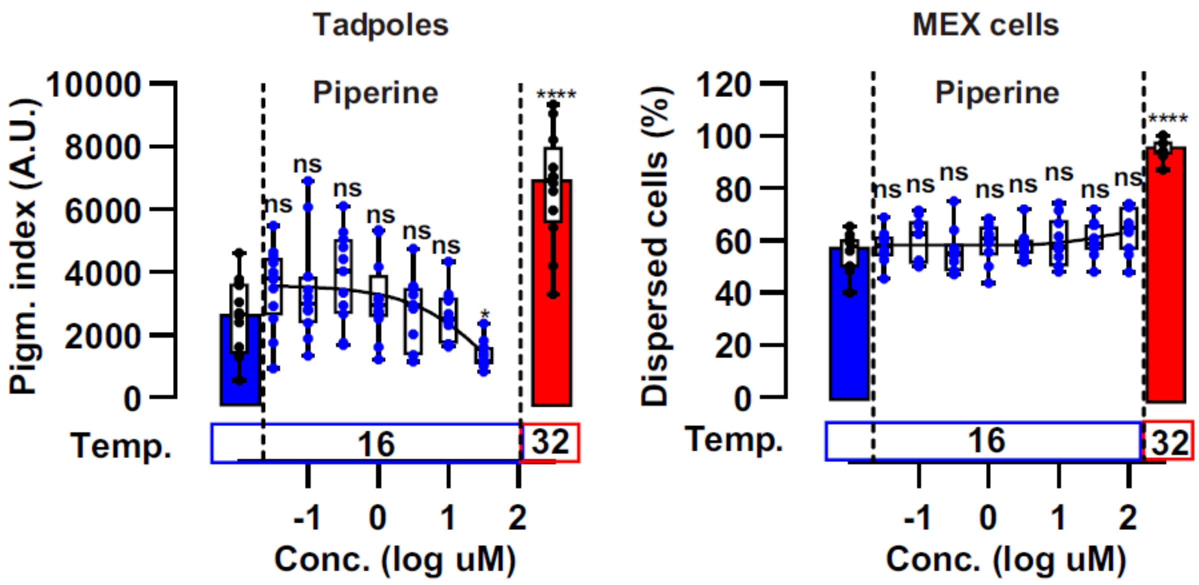

Supplementary Figure 1: Tadpoles (Stage 43/44) or MEX cells were treated with piperine. The agonists were added at 16 °C and maintained at that temperature for 45 minutes. Data points (n=9 embryos or n=6 MEX cells pictures) and a box plot (25th to 75th percentile) are represented; A representative study from three (N=3) independent experiments is shown. Statistical significances are indicated against the control without the drug at the same temperature. ns; nonsignificant. *; p<0.05. ****; p<0.0001.

Supplementary Table 1: Primers used to clone *trpv* and *trpa* genes (All oligonucleotides were obtained from INTEGRATED DNA TECHNOLOGIES)

Position in L	gene bank #	Position in S	gene bank #	sequence	primer identity (%) to L or S	PCR product cloned Size / name
trpv1.L/S F1498	XM_041581644.1	F1472	XM_041582945.1	gttctgcatgccctggtagac	95	660 / *trpv1*.L
trpv1.L/S R2158		R2132		tgtcagcaagcaaagccttca	100	
trpv2.L/S F790	XM_041581649.1	F1092	XM_018248812.2	ctgctgaaggccatgtggaat	100	655 / *trpv2*.L
trpv2.L/S R1445		R1747		gggccataagtccattccgtg	95	
trpv4.L/S F1030	XM_018261018.2	F1048	XM_018244027.2	ggcggcaaaaatgacaccatccc	95	740 / *trpv4*.L
trpv4.L/S R1768		R1786		gcatctcatggcggttctcaa	100	
trpv4l.1/2.L F1214	XM_018268227.2	F819	XM_018268226.2	atgttcatgctcaggcctgtg	100	964 / *trpv4l*.1.L
trpv4l.1/2.L R2177		R1848		aggaccagggcaatcaccata	100	
trpv3.L/S F653	XM_018245670.2	F612	XM_041583285.1	attgatgtacgggctcaggga	95	484 / *trpv3*.L
trpv3.L/S R1137		R1096		gagtttggccagcaggtatct	95	
trpv5.L F522	XM_018224919.2			ctggtgagactgctctgcatg		696 / *trpv5*.L
trpv5.L R1217				tatcaaggatccgtcgtgcct		
trpv6.L F720	XM_018224923.1			gcacatcctggttctacagcc		744 / *trpv6*.L
trpv6.L R1463				ccgtctgtgttagtgagtcgc		
trpa1.L/S F965	XM_041566492.1	F950	XM_018223798.2	gcattttgccgctacacaagg	100	427 / *trpa1*.L
trpa1.L/S R1391		R1376		atggagaggggtacagccttc	95	
